# Draft Genome Sequence of Weissella soli Strain DB-2, Isolated from Nukadoko

**DOI:** 10.1128/MRA.01160-21

**Published:** 2022-01-27

**Authors:** Daisuke Fukuda, Cirilo Nolasco-Hipolito

**Affiliations:** a Medical Affairs, Merck Research Laboratories, MSD K. K., Chiyoda-ku, Tokyo, Japan; b Institute of Biotechnology, Universidad del Papaloapan Campus Tuxtepec, Tuxtepec, Oaxaca, Mexico; University of Southern California

## Abstract

Weissella soli strain DB-2 is a lactic acid bacterium that was isolated from nukadoko in Japan. We report the draft genome sequence of Weissella soli strain DB-2 to determine the presence of the genes responsible for exopolysaccharide biosynthesis, with the aim of further probiotic evaluation.

## ANNOUNCEMENT

*Weissella* is a Gram-positive, catalase-negative, non-endospore-forming lactic acid bacterium (LAB) (1, 2). Weissella soli strain DB-2 was isolated from fermented nukadoko collected in Yokohama, Japan (35.404295°N, 139.597863°E). It is reported that *Weissella* strains produce exopolysaccharides (EPS) (3). EPS possess various health benefits for the human hosts (4–6). To determine whether the strain has the genes responsible for EPS biosynthesis, we analyzed the genome sequence of the W. soli DB-2 strain.

To isolate the DB-2 strain, serially diluted nukadoko with 0.8% NaCl solution was spread onto MRS agar (Difco) and incubated at 30°C for 2 days. The catalase-negative colonies were screened to select the LAB strain using a previously described method (7); thus, the DB-2 strain was obtained among them. The 16S rRNA gene of DB-2 was amplified by colony PCR using the primers 27F and 1492R (8) and then was directly sequenced. The sequence was subsequently analyzed using the NCBI BLAST server, which revealed its closest relatedness to Weissella soli Mi268^T^ (GenBank accession number AY028260), with an identical 16S rRNA gene sequence.

A single colony of strain DB-2 was cultured in MRS broth (Difco) at 30°C for 15 h. Then, genomic DNA was extracted and purified using the GeneJET genomic DNA purification kit (Thermo Fisher Scientific), following the instruction manual. After the quality and quantity of the genomic DNA obtained were checked using a Synergy LX microplate reader (BioTek, USA) and the QuantiFluor double-stranded DNA (dsDNA) system (Promega), respectively, the sequence library was prepared using a Nextera XT library preparation kit (Illumina). The quantity and quality of the library were verified using the Agilent fragment analyzer and the dsDNA 915 reagent kit (Advanced Analytical Technologies). Whole-genome sequencing was performed using the DNBSEQ-G400 sequencer with 2 × 200-bp paired-end reads. The sequences were processed to remove low-quality bases using Cutadapt v. 1.9.1 (9) and Sickle v. 1.33 (https://github.com/najoshi/sickle) with default parameters to obtain clean data. Sequencing resulted in 6,533,071 paired-end reads. *De novo* genome assembly was performed using the SPAdes v. 3.15.3 genome assembler (10) with default parameters. The assembly yielded 30 contigs covering a total of 1,673,321 bp, with an *N*_50_ value of 175,759 bp, a G+C content of 43.5%, and 1,562× genome coverage. The genome sequence was annotated using the DFAST v. 1.14.5 annotation pipeline (11, 12), which revealed 1,581 coding DNA sequences (CDSs), 58 tRNA genes, 2 rRNA genes, 3 CRISPR regions, and a coding proportion of 87.1%. The average nucleotide identity (ANI) was analyzed using the JSpeciesWS online service (13), which showed 98.9% and 79.4% identities with the genomes of W. soli CCUG 46608^T^ (GenBank accession number GCA_012396465.1) and Weissella koreensis KCTC 3621^T^ (GenBank accession number GCA_000277645.1), respectively. These ANI values confirmed that strain DB-2 belongs to the species W. soli.

Four genes encoding putative EPS biosynthesis proteins, including *epsB*, *epsC*, and *epsD* genes, were identified in the genome of W. soli DB-2. These homologous genes were also identified in Lactococcus lactis NIZO B40 (14).

### Data availability.

The genome sequence was deposited in DDBJ/ENA/GenBank under the accession number BQJZ01000000.1. The draft genome project data have been submitted under BioProject accession number PRJDB12623, DRA accession number DRA012883, and Sequence Read Archive (SRA) accession number DRX311665.
